# Hematometrocolpos following Low Transverse Cesarean Delivery Complicated by Uterine Dehiscence

**DOI:** 10.1155/2021/5591893

**Published:** 2021-06-29

**Authors:** Lauryn C. Gabby, Katherine E. McDaniel, Brian J. Gordon, Laila A. Al-Marayati

**Affiliations:** ^1^Department of Obstetrics, Gynecology, & Reproductive Sciences, University of California, San Diego, California, USA; ^2^Department of Obstetrics and Gynecology, University of Southern California, Los Angeles, California, USA

## Abstract

**Background:**

Hematometrocolpos is a rare complication following procedures performed on the female genital tract. While usually seen in adolescents with congenital anomalies including imperforate hymen and vaginal stenosis, it has also been described following obstetric vaginal lacerations. The incidence following cesarean delivery is unknown.

**Case:**

This is a 43-year-old multigravida who underwent a low transverse cesarean delivery complicated by uterine dehiscence, as well as cervical and vaginal lacerations. The repair resulted in lower genital tract obstruction. She presented seven months afterwards with severe abdominopelvic pain and secondary amenorrhea, which resolved after vaginal dilation and excision of the vaginal scar.

**Conclusion:**

Systematic inspection of the upper vagina should be undertaken following complicated cesarean delivery with vaginal extension. Hematometrocolpos after cesarean delivery should be managed similar to a transverse vaginal septum.

## 1. Introduction

Iatrogenic cases of hematometrocolpos are rare but have been reported after radiation therapy [1, 2] and vaginal deliveries causing obstetric trauma resulting in vaginal/labial adhesions [1–3]. Few cases of hematometra following cesarean section have been reported in the literature [4–7]; however, hematometrocolpos following cesarean section has not been yet been described, and the incidence is unknown. Most cases of hematometrocolpos are seen in adolescents, as a result of abnormal development of the lower genital tract. The most common congenital anomaly resulting in hematometrocolopos is imperforate hymen; however, transverse vaginal septum and agenesis of the lower vagina may also be etiologies of this pathology. Amenorrhea in the postpartum period is common, occurring most frequently secondary to lactation. Rarely, postpartum amenorrhea may be associated with pituitary dysfunction following postpartum hemorrhage or intrauterine adhesions secondary to pregnancy-related curettage. Here, we describe a case of postpartum amenorrhea and severe hematometrocolpos as a result of vaginal and cervical laceration repair during a low transverse cesarean delivery.

## 2. Case

This patient is a 43-year-old gravida 5, para 4, and abortus 1 with a history of uterine fibroids and two prior cesarean sections. The patient was referred by her obstetrician to Los Angeles County-University of Southern California (LAC+USC) Medical Center in 2019 for increasing pelvic pain and amenorrhea for seven months after her second cesarean delivery. Upon presenting to our institution, she reported that her pain was severe and had been gradually worsening over time with increasing intensity five weeks prior to presentation. The patient described the pain as constant and dull, localized to the pelvis and radiating to the lower back. Her pain was initially controlled with ibuprofen; more recently, she had required opioid medications for adequate pain control. Prior to receiving care at LAC+USC, the patient was initially evaluated for pelvic pain five months following her cesarean delivery in an emergency department. No imaging was performed at that time, and the provided diagnoses were uterine fibroids and incisional scarring. The patient then presented to other urgent care settings for worsening pain. At her first urgent care visit, she was treated empirically for endometritis without improvement in her symptoms. At another urgent care visit, a computed tomography (CT) scan of the abdomen and pelvis was performed and revealed an enlarged uterus with multiple fibroids and an enlarged cavity with suspicion of hematometra. She then presented to an outside outpatient clinic for follow-up and a stenotic cervical os was visualized on exam; she was subsequently referred to our institution.

The cesarean delivery operative report was obtained and revealed that the cesarean delivery was performed for persistent category two fetal heart rate tracing in the setting of one prior cesarean delivery. Upon entry into the abdomen, the surgeon documented a uterine scar dehiscence and multiple fibroids. The location and size of the fibroids were not described. After delivery of the infant, multiple supracervical and vaginal sulcal lacerations were encountered. These were repaired abdominally with a running 0-vicryl suture, beginning at the apex of the tear and suturing towards the hysterotomy. No other complications were noted. The patient was subsequently discharged home on postoperative day number two after meeting all postoperative milestones. At her postpartum visit, her provider documented her exam as limited due to an enlarged uterus and uterine fibroids. She ceased breastfeeding after two months and had not been using any hormonal or other methods of contraception at the time she was first seen at our institution.

**Figure 1:**
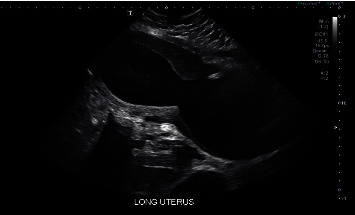
Transabdominal sagittal view of the uterus and vagina. The vagina, cervix, and uterus are distended with heterogeneous fluid.

**Figure 2:**
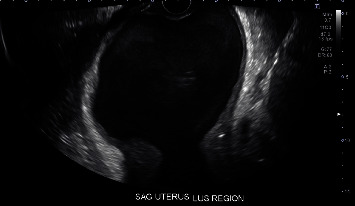
Transvaginal sagittal view of the vagina, cervix, and lower uterine segment.

**Figure 3:**
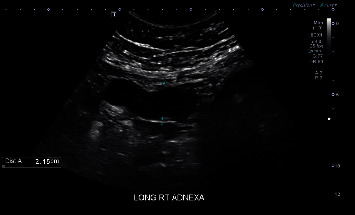
Transabdominal coronal view of the right adnexa showing a hypoechoic structure, suggestive of hydrosalpinx or hematosalpinx.

Upon presentation to LAC+USC Medical Center, she had no urinary symptoms, and urinalysis was negative. A pelvic exam revealed an enlarged and tender uterus, approximately 22 weeks in size. Digital exam revealed a likely blind ending of the vaginal canal. The cervix was not palpated on exam nor clearly visualized on speculum exam. Transvaginal and transabdominal ultrasound were performed. The uterus measured 21.8 cm × 9.4 cm × 1.3 cm (Figure 1). The ultrasound showed a heterogeneous fluid-filled upper vagina and endometrial canal (Figure 2), as well as hypoechoic structures in the adnexa suggestive of hydro/hematosalpinges (Figure 3). Overall, the ultrasound findings were concerning for hematometrocolpos.

Due to her severe pain and suspected outflow tract obstruction, the patient was taken urgently to the operating room. During exam under anesthesia (EUA), a bulging obstruction was visualized within the upper vagina. An indentation was identified and a small opening created with a scalpel. This opening was then expanded using Hegar dilators under transabdominal ultrasound guidance with efflux of a large volume of old blood. Transabdominal ultrasound then revealed a persistent fluid collection above the level of dissection. A vaginoscopy was performed with passage of the hysteroscope through the narrow opening that had been entered with the Hegar dilators. Multiple pockets of fluid were serially identified, probed gently with the Hegar dilators, and then drained. Although visualization of the portio vaginalis was suboptimal, the cervix was deemed to be dilated and without stenosis. Vaginoscopy and ultrasound revealed that there was a small fluid collection contiguous with the uterine cavity above the level of the cervix, but no further blood was noted to be draining. The procedure was then terminated. Immediately postoperatively, the patient reported improvement in her pain and was discharged home the same day. She was seen in clinic two weeks later, where she reported improvement in her symptoms. However, on sterile speculum exam, she was again noted to have a bulge in the upper vagina. Ultrasound confirmed a persistence of her hematometrocolpos. She was therefore offered planned repeat vaginal exploration with scar excision versus hysterectomy and upper vaginectomy. She elected for scar excision.

The patient subsequently returned to the operating room and underwent an EUA, cystoscopy, vaginoscopy, and excision of vaginal scar. EUA and cystoscopy were performed first for surgical planning. The cystoscopy showed no fistulae or masses. Attention was then turned to the vagina, where the vaginal scar was palpated, and a small cruciate incision was made in the midline with a scalpel with efflux of a moderate volume of old blood products. This opening was serially expanded with Hegar dilators followed by placement of a 30 cc Foley balloon over a catheter guide. The scar was then placed on tension and estimated to be about 2 cm in thickness. The vaginal epithelium proximal to the scar was undermined, and the scar tissue was removed circumferentially by dividing it into quadrants, until the dilated cervix was visible. The denuded vaginal tissue at the base of the scar was covered by reapproximating the distal vaginal epithelium to the proximal vaginal epithelium in a circumferential fashion with 2-0 polyglactin suture. Intraoperative ultrasound showed complete resolution of the hematometrocolpos, and the procedure was terminated. The patient was then discharged home the same day.

She was subsequently followed in the outpatient setting. The patient had return of regular menses and complete resolution of her pain within two months of the surgery. She reported continued normal menses seven months after the scar revision.

## 3. Discussion

Hematometrocolpos is a very rare complication following cesarean delivery, with only a few reports of hematometra existing in the literature [4–7]. Our patient experienced cervical and vaginal lacerations during the cesarean procedure, which were repaired abdominally. She subsequently experienced months of amenorrhea and worsening abdominopelvic pain. Our investigations led to a diagnosis of hematometrocolpos, which was surgically corrected with scar revision. She subsequently had return of normal monthly menses and resolution of abdominal pain.

Cases of postcesarean hematometra identified in the literature were the result of a variety of causes. One reported case of hematometra after a cesarean delivery resulted after the placement of hemostatic sutures in the lower uterine segment following delivery of a placenta previa [4]. This led to uterine synechiae which required hysteroscopic adhesiolysis. A separate case likely resulted from inadvertent suturing of the anterior and posterior uterine walls during hysterotomy repair, which created a uterine pouch that did not communicate with the vagina [5]. Uterine compression sutures and postoperative endometritis have also been associated with postcesarean hematometra [6, 7].

Our patient's main risk factors for this outcome were a history of prior cesarean delivery, uterine dehiscence, and extensions of the hysterotomy into the vagina. The complex nature of the initial vaginal lacerations may have led to inadvertent closure of the vagina and/or the formation of significant scar tissue resulting in hematometrocolpos. In order to reduce the risk of postoperative persistent lower genital tract obstruction after extensive vaginal laceration repair at the time of delivery, the patency of the vagina may be assessed either by speculum exam or with digital palpation of the uterine cervix. Intraoperative assessment of the upper vagina at the time of cesarean delivery could reduce the risk of iatrogenic occlusion, though the chance of upper vaginal scarring may not be completely eliminated. Additionally, a thorough pelvic exam at the time of the postpartum visit following a cesarean delivery may be performed for complete evaluation of the lower genital tract. Heightened awareness of this potential postoperative complication and prompt investigation with imaging may lead to earlier recognition and surgical correction, which could greatly reduce morbidity. Furthermore, lower genital tract obstruction should always be included in the differential for postpartum amenorrhea, especially in patients that are no longer breastfeeding. Lastly, it is important to note that our initial surgical approach—vaginal dilation of the patient's genital tract obstruction—was not successful. As a result, we suggest first attempting surgical correction with scar excision in a manner similar to the approach for a transverse vaginal septum.

Although hematometrocolpos is a rare complication of cesarean delivery, it is important to identify potential risk factors for lower genital tract obstruction during and after the delivery and to treat with prompt surgical excision of the obstruction.

## Data Availability

Not applicable as this is a case report.
